# Robotic crabs reveal that female fiddler crabs are sensitive to changes in male display rate

**DOI:** 10.1098/rsbl.2017.0695

**Published:** 2018-01-17

**Authors:** Sophie L. Mowles, Michael D. Jennions, Patricia R. Y. Backwell

**Affiliations:** 1 Department of Biology, Anglia Ruskin University, Cambridge CB1 1PT, UK; 2 Ecology & Evolution, Research School of Biology, The Australian National University, Canberra, ACT 0200, Australia

**Keywords:** courtship, fiddler crab, mate choice, robotic playback, stamina, *Uca mjoebergi*

## Abstract

Males often produce dynamic, repetitive courtship displays that can be demanding to perform and might advertise male quality to females. A key feature of demanding displays is that they can change in intensity: escalating as a male increases his signalling effort, but de-escalating as a signaller becomes fatigued. Here, we investigated whether female fiddler crabs, *Uca mjoebergi*, are sensitive to changes in male courtship wave rate. We performed playback experiments using robotic male crabs that had the same mean wave rate, but either escalated, de-escalated or remained constant. Females demonstrated a strong preference for escalating robots, but showed mixed responses to robots that de-escalated (‘fast’ to ‘slow’) compared to those that waved at a constant ‘medium’ rate. These findings demonstrate that females can discern changes in male display rate, and prefer males that escalate, but that females are also sensitive to past display rates indicative of prior vigour.

## 1. Introduction

Dynamic, repeated displays are often performed by males during courtship interactions and occur in several modalities [1]. Repetition of dynamic courtship signals can be energetically costly and thereby reveal the quality of the signalling male [1,2]. For example, courtship can cause male field crickets (*Gryllus bimaculatus*) to undergo anaerobic respiration [3] and male fiddler crabs (*Uca mjoebergi*) demonstrate a prolonged reduction in sprint performance post-courtship, indicative of lactic acid build-up [4] and oxygen debt [5]. This heavy investment in signal production is likely to allow females to select physically fit mates as these ‘signals of stamina’ will reflect a male's ability to perform other demanding activities associated with survival [6], and reduce the risk of mating with weaker signallers that might be diseased or parasitized [7].

In addition to the potential for high intensity signalling to increase signal efficacy, females should be able to select physically fit males by attending to their display rate. Indeed, the females of many species demonstrate preferences for males that perform high intensity courtship signals. For example, female fiddler crabs generally prefer males that wave at higher rates than their rivals [8]. However, a characteristic of dynamic, repeated displays is that the rate of display changes during the course of an interaction. This is especially true of energetically costly signals [1,2], because a signaller often initiates a display with a low intensity signal to avoid unnecessary production costs, but increases his signalling effort if the courted female needs more inducement to mate. Thus, energetically costly signals can escalate in intensity throughout an interaction, terminating at the signaller's energetic cost threshold if the female has not yet made the decision to mate. Equally, energetically costly signals can de-escalate if the signaller approaches its cost threshold and succumbs to fatigue. Thus, females should not only attend to the absolute, current level of courtship signal production, but also to any changes in rate that might provide more accurate information about a signaller's quality.

Here, we address whether females attend to changes in courtship rate in the fiddler crab. Males have one greatly enlarged claw that is used in a courtship waving display [9]. We presented females with replica robot males that waved in the species-specific pattern (see [10]) at either a constant rate, or at a rate that escalated or de-escalated as the encounter progressed.

## 2. Material and methods

We carried out fieldwork from November to December 2014 at East Point Reserve, Darwin, Australia (12°24′32″ S; 130°49′50″ E) during the diurnal low tide period of neap tides. We collected wandering female *Uca mjoebergi*, usually indicative of mate searching [11], and placed them individually in plastic cups filled with 1 cm of seawater. These were kept in the shade until they were used in the mate-choice trials.

Experiments were conducted using identical robotic crab units: replica male fiddler crabs composed of an accurately painted [12] hydrostone *U. mjoebergi* claw (21.1 mm) mounted to a small robotic arm that mimics the courtship wave movements [10]. Robotic crab units were inserted in a 60 × 60 cm raised platform covered in mudflat substrate. Units were 5 cm apart, placed 20 cm from the release mechanism, and orientated to face the female, which was placed under a small transparent plastic container that was remotely released (see electronic supplementary material, figure S1). Females could thus see the waving sequence of the robots from its initiation prior to their release. Each robotic male was programmed to wave with one of three patterns: escalating, constant and de-escalating. The escalating robot started at a ‘slow’ rate of 3.95 waves min^−1^ and gradually increased to a ‘fast’ rate of 15.79 waves min^−1^ over a 90 s period, after which it continued at the ‘fast’ rate. The constant robot waved at a constant ‘medium’ speed of 7.90 waves min^−1^ for the entire test. The de-escalating robot started at a ‘fast’ rate of 15.79 waves min^−1^ and gradually decreased to a ‘slow’ rate of 3.95 waves min^−1^ over a 90 s period, after which it continued at the ‘slow’ rate.

Females were used in three treatments:

1. Escalation choice trials (*N* = 40 females): female presented with two robots, one escalating and one waving at a constant rate.
2. De-escalation choice trials (*N* = 40 females): female presented with two robots, one de-escalating and one waving at a constant rate.
3. Three-choice trials (*N* = 65 females): female presented with three robots, one escalating, one de-escalating and one waving at a constant rate.

**Figure 1.**
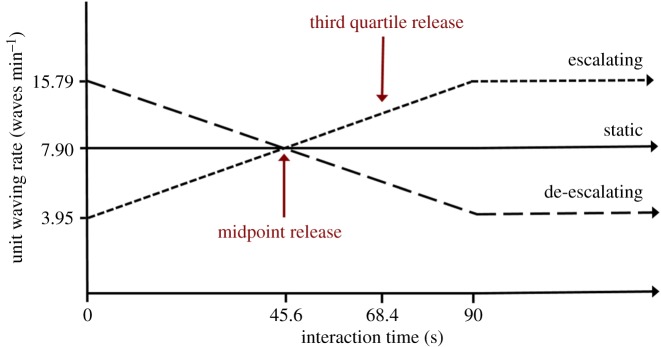
Timeline illustrating the signal rates produced by each robotic unit throughout the interaction sequence. (Online version in colour.)

Each female was used in one of the three treatments and twice during this treatment [10], being released at two time points: Release 1 = halfway through the sequence (45.6 s into interaction time), when all robots simultaneously waved at the same rate (figure 1). Release 2 = three quarters of the way through the sequence (68.4 s into interaction time), when the escalating or de-escalating robots had begun to approach their final wave rate (figure 1). Half of the females experienced Release 1 first and half experienced Release 2 first, with a rest period in between.

A choice was recorded if the female contacted the robotic crab and her latency to choose was recorded in seconds. Trials in which the female displayed a startle response, left the arena or did not choose within 180 s were eliminated. After testing, females were placed in a new burrow on the mudflat.

Female preferences for the robotic males were tested using *χ*^2^ tests, while female choice latencies were compared using Wilcoxon rank sum tests and Kruskal–Wallis rank sum tests in R v. 3.4.1.

## 3. Results

**Figure 2.**
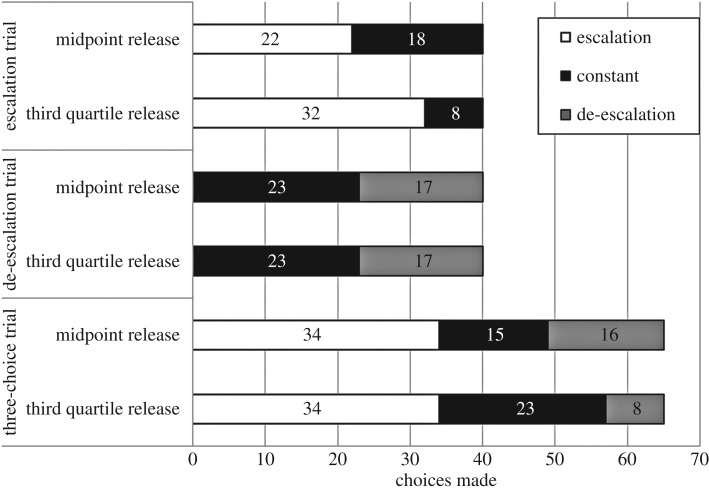
Choices made by females at each of the two time releases in each of the three treatments: escalating versus constant robot; de-escalating versus constant robot; and escalating versus constant versus de-escalating robots.

In the two-choice trials, there was no female preference for either robot in the escalation (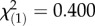, *p* = 0.527, *N* = 40) or in the de-escalation choice trials (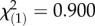, *p* = 0.343, *N* = 40) when females were released mid-way through the wave sequence. However, when released three-quarters of the way through the sequence, females significantly preferred the escalating robot (
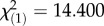
, *p* = 0.0001, *N* = 40), but, again, showed no preference for either robot in the de-escalation choice trials (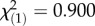, *p* = 0.343, *N* = 40; figure 2). There was no difference in the latency to choose between the robots chosen (see electronic supplementary material, table S1).

When females were presented with an escalating robot, a de-escalating robot and one that waved at a constant rate, they exhibited a significant preference for the escalating robot compared to either the constant rate robot (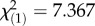, *p* = 0.007, *N* = 49) or the de-escalating robot (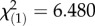, *p* = 0.011, *N* = 50) when released mid-way through the wave sequence. They did not discriminate between the constant and de-escalating robots (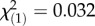, *p* = 0.858, *N* = 31; figure 2). There was no difference in the latency to choose between the robots chosen (see electronic supplementary material, table S1).

Females showed a similar set of preferences when they were released three-quarters of the way through the wave sequence (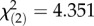, *p* = 0.114, *N* = 130). They significantly preferred the escalating robot (
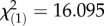
, *p* < 0.0001, *N* = 42) and the constant wave robot (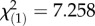, *p* = 0.007, *N* = 31) over the de-escalating robot. They did not, however, discriminate between the escalating and constant wave robots (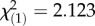, *p* = 0.145, *N* = 57). Here, decision times differed between the three robotic males, with females that took longer to decide being more likely to choose the escalating robot (see electronic supplementary material, table S1).

## 4. Discussion

In two-choice trials, female *Uca mjoebergi* showed no preference for escalating or de-escalating robots over those with a constant wave rate when all robots were simultaneously waving at the same rate at the time of release. However, females significantly preferred robots with an escalating wave rate when released later in the interaction sequence. This could demonstrate that females responded to the *current* signal rate. It has been previously demonstrated that female fiddler crabs prefer males that signal at a higher rate [8], perhaps because signalling rate is associated with performance capacities, which are indicative of male quality [5]. However, if females simply choose males based on their *current* display rate, then we would also expect them to significantly prefer males signalling at the constant rate over the de-escalating rate when released later in the sequence, as the former have a higher current wave rate. We would also expect to see differences in decision latencies between the choices, with longer latencies resulting in fewer females choosing the de-escalating robots, which would have become slower. Yet this was not the case. There are two plausible explanations. (1) Females selecting the de-escalating male remembered his earlier wave rate and assessed that he had signalled vigorously at the start of the interaction. (2) Females have a threshold wave rate above which a choice decision is triggered. The medium and slow wave rates, if below this threshold, would not elicit a preference.

The final trials that involved three robots with an escalating, de-escalating or constant wave rate might allow us to distinguish between these competing explanations. For the mid-trial releases, females exhibited a significant preference for the escalating robot over both the constant rate and de-escalating robots. This demonstrates that females are sensitive to changes in rate and that when signal rates are perceptibly changing among the males in a group, females select the ones that are escalating, even when choosing at the point at which all robots were simultaneously waving at the same rate. Such males might have greater motivation to court, and might be on a trajectory to increase their wave rate further, while also demonstrating that they can conserve energy until necessary. However, once the wave sequence has progressed, females with a greater latency to choose selected the escalating robot, having gathered more information about his increasing wave rate. Females also exhibited a significant aversion to the de-escalating robot, while choosing evenly between the robots that either escalated or waved at a constant rate. Although females detected that the de-escalating robot was slowing, hence avoided it, the lack of discrimination between the other two males suggests that they made a quick, error-prone final decision. This might be because predators are a greater risk in the presence of multiple signalling males [13]. Nonetheless, the clear aversion to the de-escalating robot at this point demonstrates that females are capable of resolving the differences between a ‘medium’ and ‘slow’ wave rate. This implies that when females exhibited no preference in the two-choice de-escalation trials, they could discern the wave rate differences and based their decisions on the prior rather than current display rates.

Changes in display rate are important to how animals signal. Energetically costly dynamic repeated displays are likely to escalate when a male attempts to persuade a female to mate by increasing his signalling effort, but can eventually de-escalate as he becomes fatigued [1,2]. Females should be sensitive to these rate changes as they could indicate that a male has greater signalling capacity than initially advertised, or that a male, despite appearing to be a vigorous and effective signaller, has exhausted his energetic reserves in what would effectively be an unreliable signal (see [5]). Further, in species such as fiddler crabs, where the male bears a formidable weapon, signalling rate could also indicate motivation to court, where males expending energy in a costly display are less likely to react with dangerous levels of aggression towards approaching females. As in fiddler crabs, females of many species may be sensitive to changes in display rate and benefit from attending to prolonged dynamic repeated courtship displays, which provide more reliable information with which to accurately gauge male quality.

## Supplementary Material

ESM Figure 1

## Supplementary Material

ESM Table 1

## Supplementary Material

Robotic crab choice data

## Data accessibility

The dataset supporting this article has been uploaded as part of the electronic supplementary material.
