# Supplementary figures and images for: Robotic crabs reveal that female fiddler crabs are sensitive to changes in male display rate
Source: Biol Lett. 2018 Jan 17;14(1):20170695. doi: 10.1098/rsbl.2017.0695 (PMC5803598; doi:10.1098/rsbl.2017.0695)

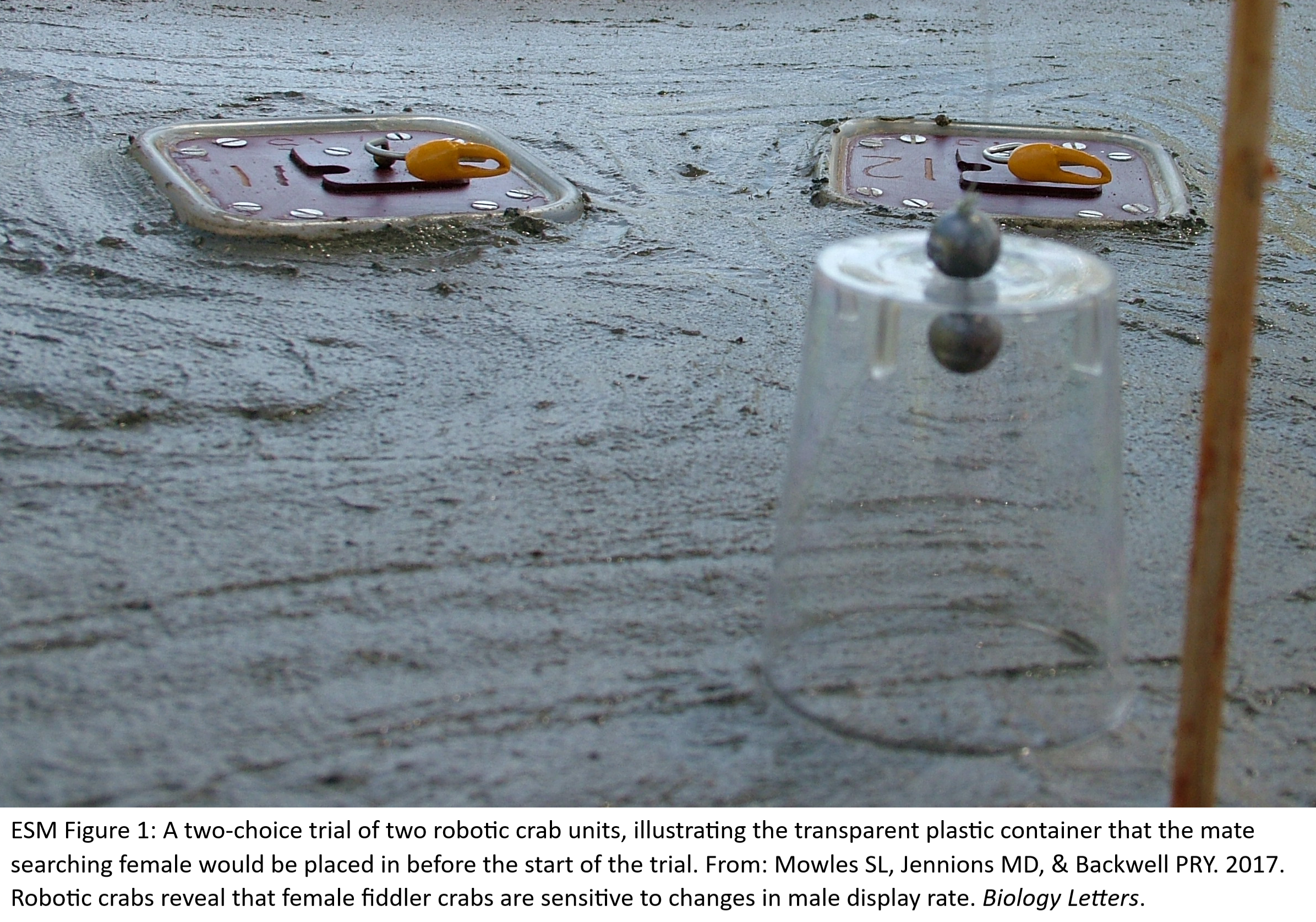

Supplement: ESM Figure 1 [file rsbl20170695supp1.tiff]
