# Supplementary material for: Robotic crabs reveal that female fiddler crabs are sensitive to changes in male display rate
Source: Biol Lett. 2018 Jan 17;14(1):20170695. doi: 10.1098/rsbl.2017.0695 (PMC5803598; doi:10.1098/rsbl.2017.0695)
Supplement: ESM Table 1 [file rsbl20170695supp2.docx]

ESM Table 1. Latencies of females to choose at each of the two time releases (Midpoint = 45.6s, Third Quartile = 68.4s) in each of the three treatments. From: Mowles SL, Jennions MD, & Backwell PRY. 2017. Robotic crabs reveal that female fiddler crabs are sensitive to changes in male display rate. Biology Letters.

| **Escalating trial** | Median (s) | *W* | N | *P* |
| --- | --- | --- | --- | --- |
| Midpoint release | Escalating: 29.39  Constant: 16.79 | 269.5 | 40 | 0.054 |
| Third quartile release | Escalating: 14.03  Constant: 12.83 | 138 | 40 | 0.748 |
| **De-escalating trial** | Median | *W* | N | *P* |
| Midpoint release | Constant: 17.69  De-escalating: 16.10 | 181 | 40 | 0.705 |
| Third quartile release | Constant: 30.07  De-escalating: 24.22 | 199 | 40 | 0.935 |
| **Three choice trial** | Median | Kruskal Wallis *X*^2^_(2_*_)_* | N | *P* |
| Midpoint release | Escalating: 28.73  Constant: 16.35  De-escalating: 19.33 | 5.123 | 65 | 0.077 |
| Third quartile release | Escalating: 15.99  Constant: 9.78  De-escalating: 4.98 | 7.192 | 65 | **0.027** |
